# Rare presentation of renal cell cancer as dysphagia: a case report

**DOI:** 10.1186/s13256-018-1967-6

**Published:** 2019-03-19

**Authors:** Manmeet S. Padda, Wei M. Si

**Affiliations:** 1 Gastroenterology and Hepatology, DHAT, Scott and White Baylor Medical Center, McKinney, TX USA; 2 Department of Pathology, AmeriPath, Plano, TX USA

**Keywords:** Renal cell cancer, Esophageal metastasis, Case report

## Abstract

**Background:**

Metastasis from distal solid organs to the esophagus is very rare. Renal cell cancer with esophageal metastasis is extremely rare. We present the first case report of undiagnosed renal cell cancer presenting as dysphagia.

**Case presentation:**

A 56-year-old Caucasian man presented for dysphagia evaluation. An esophagogastroduodenoscopy examination revealed a 6 mm nodule located at gastroesophageal junction. Pathology and immunohistopathology were suggestive of metastatic renal cell cancer. Abdominal imaging revealed a large renal mass consistent with renal cell cancer. He underwent left nephrectomy and is clinically asymptomatic, while being monitored by Oncology and Urology.

**Conclusions:**

Undiagnosed renal cell cancer metastasis presenting as dysphagia is very rare. Careful upper endoscopy examination contributed to the diagnosis of this rare entity. A multidisciplinary team approach is key for management of these clinical dilemmas.

## Introduction

Metastasis from distant solid organs to the esophagus is very rare. We present the first case of undiagnosed renal cell carcinoma (RCC) presenting as dysphagia with metastasis to gastroesophageal junction (GEJ). Pathology from a gastroesophageal nodule warranted further workup, which led to a diagnosis of RCC. RCC metastasis after resection of primary cancer has been reported to occur in the pancreas, gastric mucosa, and duodenum. Esophageal metastasis from RCC is very rare, and has been reported after resection of primary RCC [1, 2]. A search of the PubMed database did not reveal prior publication of diagnosis of renal cell metastasis to GEJ, established prior to diagnosis of primary RCC.

## Case presentation

**Fig. 1:**
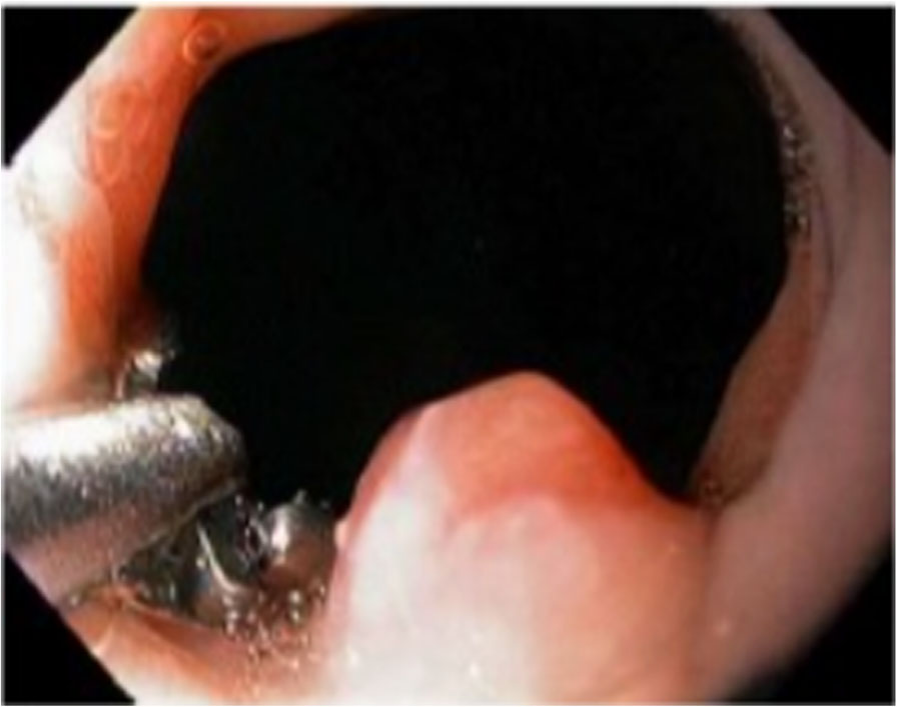
Nodule located at gastroesophageal junction

**Fig. 2:**
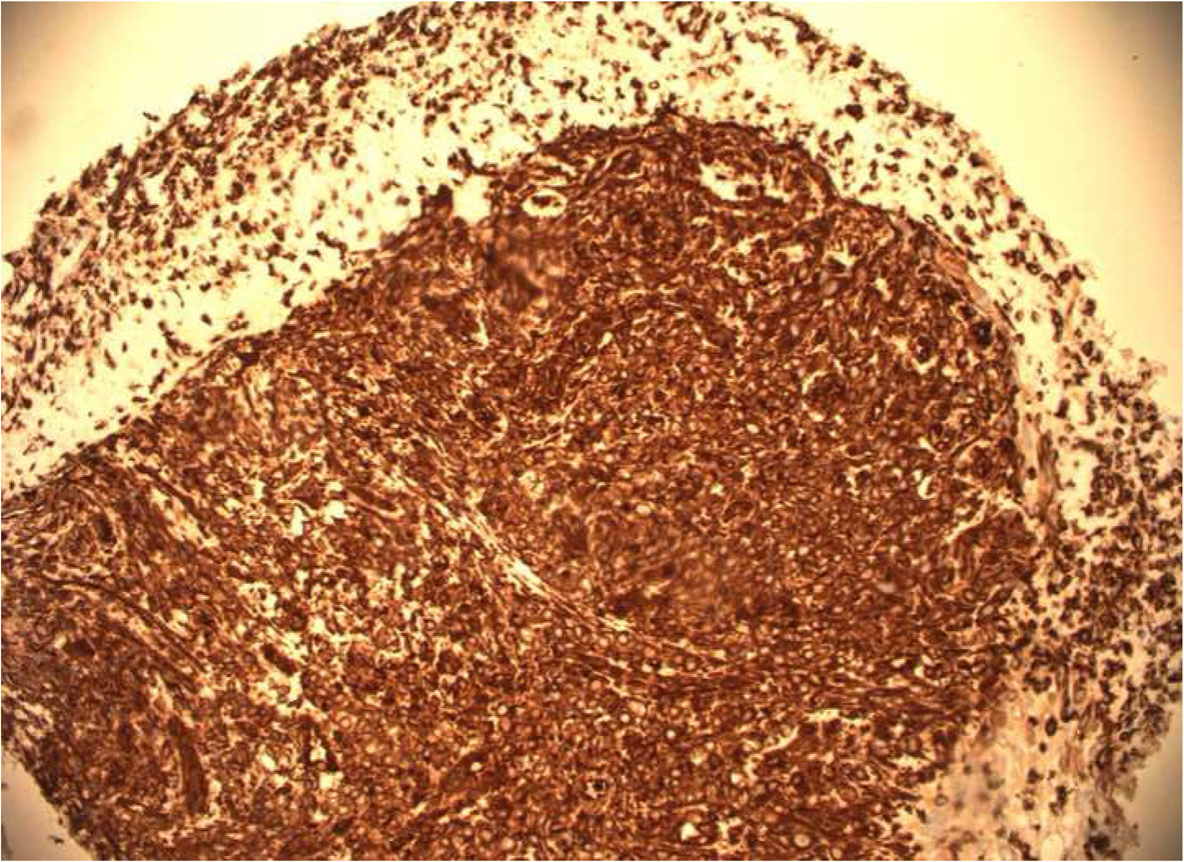
Positive vimentin immunostain

**Fig. 3:**
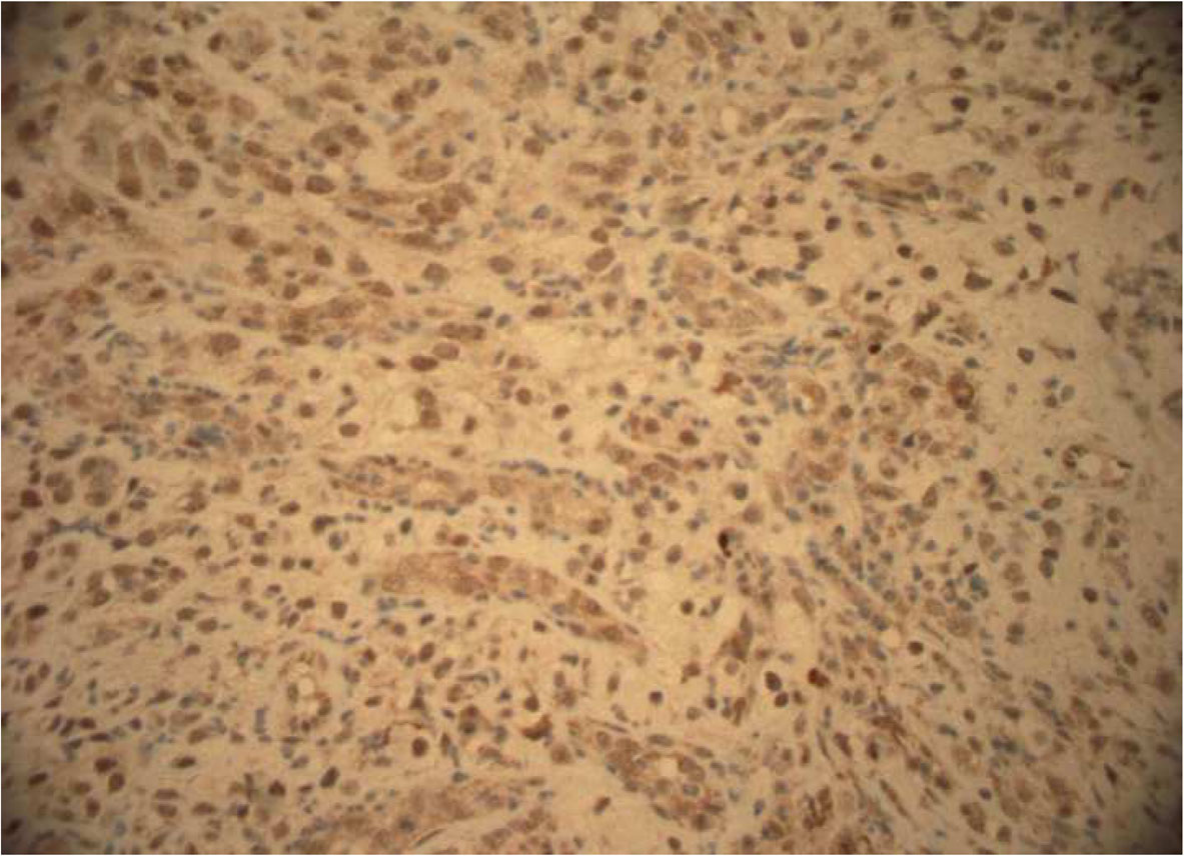
Positive PAX-8 stain

**Fig. 4:**
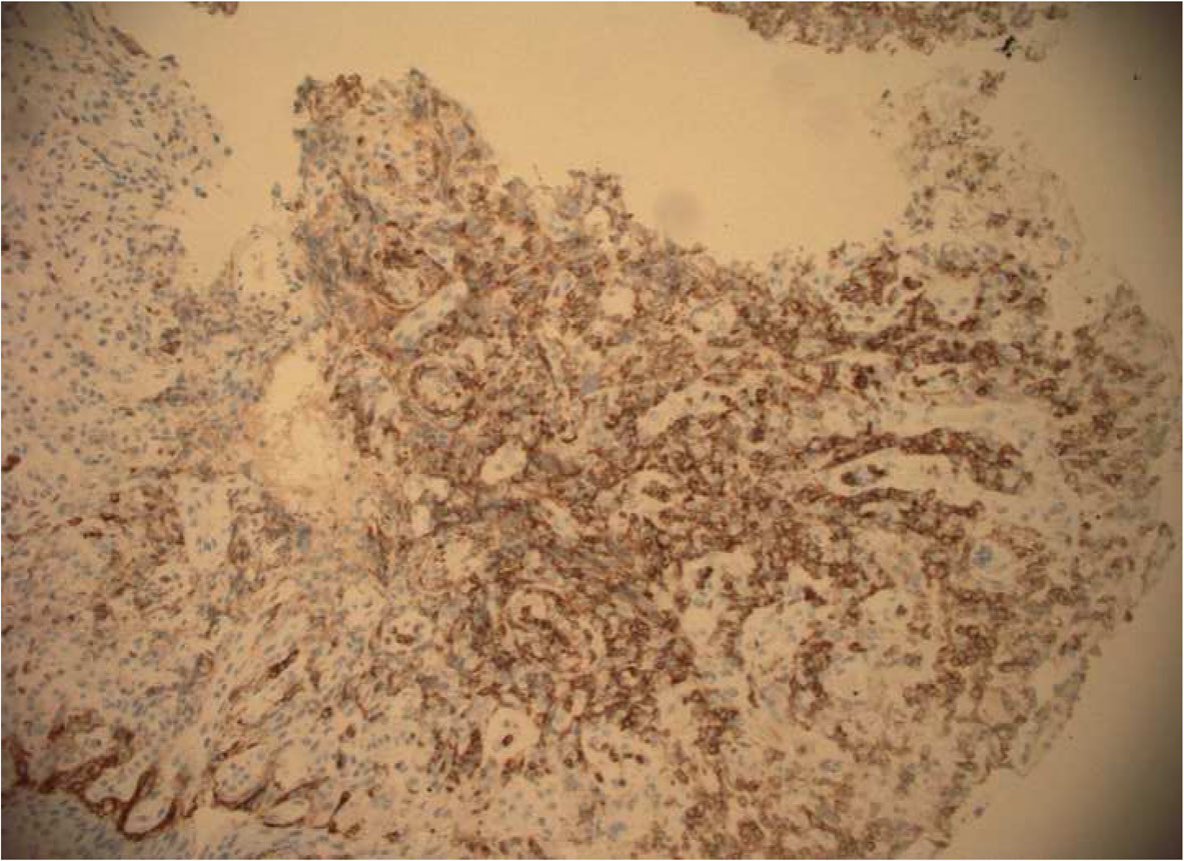
Positive CD10 stain

**Fig. 5:**
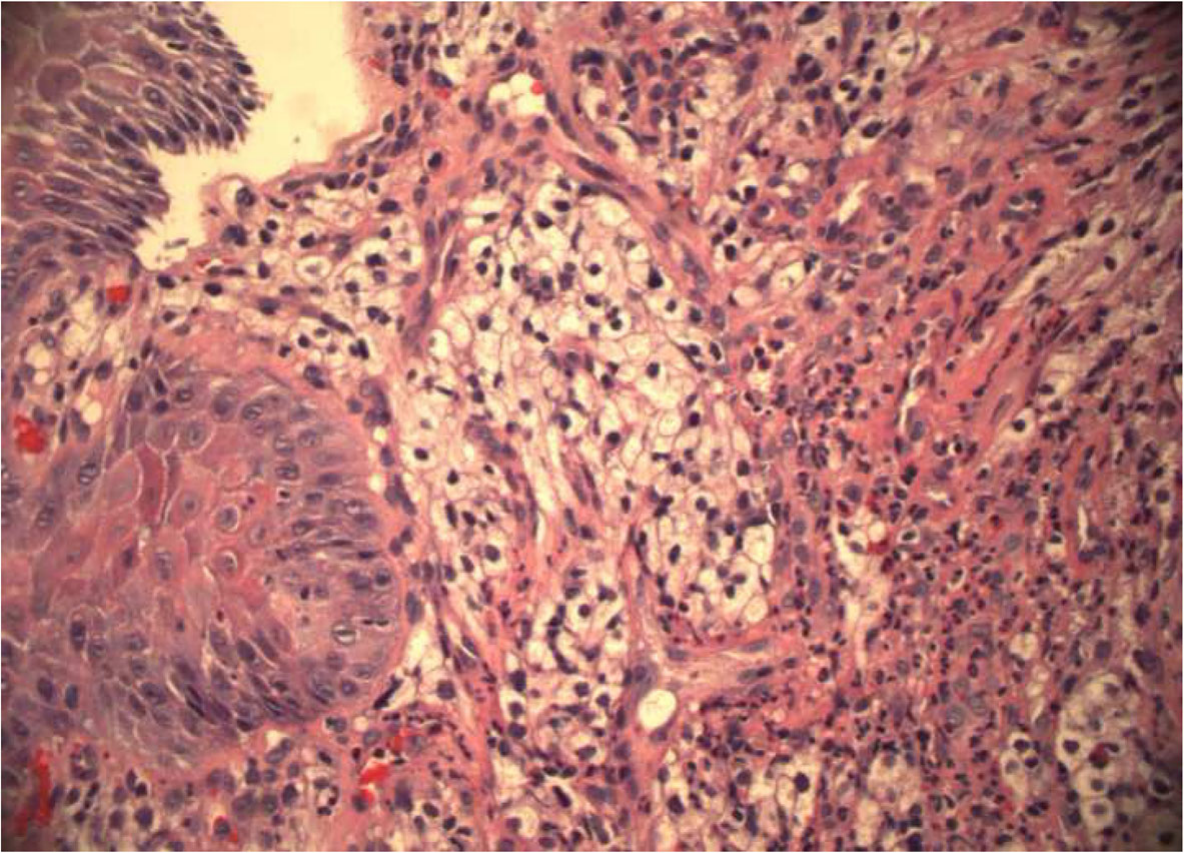
Immunohistochemistry of gastroesophageal junction nodule

A 56-year-old Caucasian man presented for evaluation of intermittent dysphagia to solids for the past few weeks. He had no significant past medical history. He had the sensation of food getting stuck in substernal area. Otherwise he reported good appetite and no weight loss. A physical examination revealed a well-nourished man with no palpable mass or lymph nodes. An abdominal examination revealed no localized tenderness or organomegaly. No family history of stomach or colon malignancy was reported. An esophagogastroduodenoscopy (EGD) examination revealed a single 6 mm nodule at GEJ (Fig. 1) and Los Angeles grade A (less than 5 mm mucosal breaks) distal esophagitis. A pathology examination from the GEJ nodule showed squamous mucosa with mucosal ulcer and associated acute and chronic inflammatory infiltrates. Nests of atypical cohesive cells with clear cytoplasm, and mild nuclear pleomorphism were noted in submucosa. Multiple immunostains were performed to further characterize atypical cells with following staining pattern: vimentin (+), PAX-8 (+), CD10 (focally and weakly positive), and epithelial membrane antigen (EMA; focally and weakly positive) (Figs. 2, 3, 4, and 5). These cells were negative for RCC, thyroid transcription factor 1(TTF1), S100 protein, CD68, cytokeratin 5/6, pancytokeratin, p63, cytokeratin 7 and 20, p40, and pan melanoma marker. Histological features and staining patterns were consistent with atypical clear cell infiltrate involving squamous mucosa, which was consistent with metastatic RCC. A computed tomography study of his chest, abdomen, and pelvis performed with orally and intravenously administered contrast showed unremarkable esophagus and no mediastinal lymphadenopathy.

A solid-appearing, partially exophytic mass involving his superior left kidney was seen. The renal mass measured 5.8 × 5.3 cm. The mass was heterogenous and displaced portions of upper pole. The mass was abutting the inferior aspect of his spleen, but a thin fat plane separating his spleen from the mass was seen. A small 0.6 cm gastrohepatic lymph node was seen which radiologically appeared benign. A positron emission tomography (PET) scan with 16 mCi of 18-flurodeoxyglucose showed a left upper pole renal mass measuring 5 cm with standardized uptake value (SUV) of 3, suspicious for renal malignancy. No other abnormal uptake was seen to suggest metastatic disease or lymphadenopathy. A pathology conference and expert opinion concluded that the GEJ nodule biopsy was consistent with metastatic renal clear cell carcinoma (Fuhrman grade 2) involving esophageal mucosa with erosion. Clear cell morphology showed sharp cell borders and lack of glands or mucin, consistent with RCC. A multidisciplinary conference recommended left renal nephrectomy. Our patient underwent left radical nephrectomy and path showed clear cell RCC, with no sarcomatoid features, Fuhrman nuclear grade 3. Resections margins were negative and no involvement of adrenal gland was noticed. Endoscopic submucosal dissection of area of GEJ nodule was performed, which showed squamous and gastric-type mucosa with chronic inflammation and underlying adipose tissue in submucosa, negative for malignancy. He has been doing well clinically for the past 16 months, after left nephrectomy. Oncology services recommended close surveillance and conservative management after long discussion with our patient and other consultants.

## Discussion

Intermittent dysphagia to solids is usually attributed to benign intraluminal disease of the esophagus. Schatzki ring, esophagitis, stricture, esophageal ulcer, hiatal hernia, and dysmotility were included in the differential list for dysphagia workup. Esophageal malignancy is suspected if the symptoms are associated with weight loss or risk factors (tobacco smoking, alcohol use, prolonged gastroesophageal reflux disease, lye ingestion) [3]. It is uncommon to have metastasis disease involving the esophagus, contributing to dysphagia. Metastasis from the breast, cholangiocarcinoma, colon, ovaries, and thyroid has been reported to involve the esophagus [4]. The mechanism of metastasis to the esophagus is either due to direct invasion from adjacent tumors or via hematogenous/lymphatic spread from distant organs. Hematogenous/lymphatic spread to the esophagus is very rare (1–1.3%) [5].

Kidneys have an abundant blood supply which increases risk of metastasis from RCC. Metastasis from RCC can involve lungs, bones, liver, brain, and local lymph nodes in 30% of patients. Esophageal metastasis from RCC is extremely rare, and there are only three case reports in PubMed using key words such as “esophageal metastasis” and “renal cell carcinoma” [2, 6, 7]. A review of these cases reports reflected that in all the cases, esophageal metastasis occurred after diagnosis of renal cell cancer had been established (range 5–10 years). The case report by de los Monteros-Sanchez *et al*. also elaborated on the occurrence of isolated esophageal metastasis presenting as dysphagia. Their patient was a 60-year-old woman who had renal cancer resected in the past [2]. After excision of esophageal metastasis, she survived for only 11 months [2]. None of the published cases in PubMed reported diagnosis of esophageal metastasis of RCC prior to identification of renal mass or cancer.

Hence we presented the first case of undiagnosed RCC presenting as dysphagia.

Careful upper endoscopy was key to the diagnosis of this rare presentation. The rate of missed esophageal lesions on upper endoscopy is around 7% [8]. Although the GEJ nodule was only 6 mm in size, biopsy of this diminutive lesion led to diagnosis of RCC. Dysphagia in previously undiagnosed RCC appears to cause mild symptoms as compared to significant symptoms in the setting of known RCC. Our patient is clinically asymptomatic 16 months after left nephrectomy, but in the case reported by de los Monteros-Sanchez *et al*., survival was only 11 months after esophageal metastasis resection in a known case of RCC [2].

Metastasis to the duodenum has been reported after resection of primary left renal cell cancer involving left adrenal. In our case, the left adrenal gland was not involved by malignancy. A radical left nephrectomy was recommended due to the overall good health of our patient and a PET scan showed localized disease. Previous data have reflected better outcome after resection of isolated renal cell cancer metastasis, so repeat EGD was performed with endoscopic mucosal dissection of the GEJ nodule, but surprisingly pathology showed no remnant of the malignant cells. Aggressive biopsies during index EGD probably resulted in complete removal of the metastatic lesion. Our patient declined any chemotherapy due to his hectic lifestyle involving frequent overseas trips. He has had no recurrence of dysphagia and follow-up PET scan showed no abnormal uptake. Patients with metastatic RCC need to be continuously monitored for any evidence of recurrence of malignancy via imaging studies. Our patient had a positive tumor PET scan and hence periodic PET scan surveillance was included in his plan of care.

## Conclusion

Esophageal metastasis from RCC is extremely rare. We reported the first case of undiagnosed RCC presenting as metastatic disease to the esophagus, presenting as dysphagia. A detailed history and careful endoscopy examination played important roles in diagnosis. High clinical suspicion and a multidisciplinary approach including a gastroenterologist and expert pathologists, urologists, surgical oncologists, and oncologists are essential for management of these cases.

## Abbreviations

EGD: Esophagogastroduodenoscopy
GEJ: Gastroesophageal junction
PET: Positron emission tomography
RCC: Renal cell carcinoma
